# Research on the needs of vocational high school students for injury prevention and emergency skills education

**Published:** 2019-08

**Authors:** Cheng XUE, Shumei WANG, XUE Cheng, WANG Shumei, YE Zhoufeng, LI Fenfen, REN Jun

**Affiliations:** ^*a*^School of Public Health, Fudan University, Shanghai , China.

## Abstract

**Background:**

Objective to investigate the needs of junior high school students for injury prevention awareness and emergency skills education, so as to provide reference basis for junior high school students to carry out injury prevention work.

**Methods:**

A total of 1079 vocational middle school students were selected by stratified cluster sampling. The content of the questionnaire includes: basic demographic information, cognition of injury-related risk factors, demand for injury prevention education, mastery of first aid skills, etc.

**Results:**

76.2% believed that there were potential risk factors leading to injury accidents in life, 397 (36.8%) chose yes, rarely, 303 (28.1%) chose yes, generally, and 122 (11.3%) chose yes, many .Among the places considered most vulnerable to injury, the top five were road/street, off-campus sports ground, business district, school and residential district, with 79.1%, 41.6%, 33.1%, 29.2% and 19.6% respectively. The potential risk factors considered by students are divided into four aspects. The proportion of vocational middle school students in the judgment and selection of potential risk factors are lack of safety awareness (54.9%) and excessive learning pressure (41.2%) respectively. Parents ignore their children (34.0%) and family relationship is not harmonious (31.3%) were the top two risk factors for potential injury to the family. The top two risk factors for potential injury in schools were bad school atmosphere (37.7%) and lack of school safety education (36.9%).For potential hazardous environmental factors, unsafe road environment (64.8%) and unsafe sports facilities (44.8%) ranked top two.89.0% of the students think it necessary to attend schools or other institutions to carry out safety education or activity, hope schools provide injury prevention education aspect, the top six were bullying (85.6%), food hygiene (80.3%), traffic injuries (74.4%), fraud (65.1%), electric shock (65.1%) and first aid (63.5%), especially the bullying of these safety education.78.9% of the students said they had received first aid training, and CPR (86.5%), first aid for drowning (49.4%), bandaging (42.2%) and hemostasis (42.0%) were the top four first aid skills of the middle school students surveyed. The most common reasons for not mastering first aid skills were forgetting after learning (33.5%) and too short training time (29.7%).

**Conclusions:**

Junior high school students need to improve their education on potential injury risk factors in life and emergency treatment in the face of injury.It is necessary to carry out injury prevention and emergency skills education among high school students in vocational schools.

**Keywords:**

Vocational high school, Injury prevention, Emergency treatment, Education, Demand

